# Assessment of patients with suspected sepsis in ambulance services: a qualitative interview study

**DOI:** 10.1186/s12873-021-00440-4

**Published:** 2021-04-09

**Authors:** Agnes Olander, Anders Bremer, Annelie J. Sundler, Magnus Andersson Hagiwara, Henrik Andersson

**Affiliations:** 1 University of Borås, PreHospen, Centre for Prehospital Research, SE- 405 30 Borås, Sweden; 2 University of Borås, Faculty of Caring Science, Work Life and Social Welfare, Borås, Sweden; 3 Linnaeus University, Faculty of Health and Life Sciences, Växjö, Sweden

**Keywords:** Ambulance clinicians, Ambulance services, Assessment, Interviews, Sepsis

## Abstract

**Background:**

The initial care of patients with sepsis is commonly performed by ambulance clinicians (ACs). Early identification, care and treatment are vital for patients with sepsis to avoid adverse outcomes. However, knowledge about how patients with sepsis are assessed in ambulance services (AS) by AC is limited. Therefore, the aim of this study was to explore the meaning of ACs’ lived experiences in assessing patients suspected of having sepsis.

**Methods:**

A descriptive design with a qualitative approach was used. Fourteen ACs from three Swedish ambulance organizations participated in dyadic and individual semistructured interviews. A thematic analysis based on descriptive phenomenology was performed.

**Results:**

AC experiences were grouped into four themes: (1) being influenced by previous experience; (2) searching for clues to the severity of the patient’s condition; (3) feeling confident when signs and symptoms were obvious; and (4) needing health-care professionals for support and consultation.

**Conclusions:**

This study indicates that several factors are important to assessments. ACs needed to engage in an ongoing search for information, discuss the cases with colleagues and reconsider the assessment throughout the entire ambulance mission. A reflective and open stance based on professional knowledge could contribute to recognizing patients with sepsis.

## Background

Sepsis is a life-threatening organ dysfunction caused by infection [1]. The number of patients with sepsis is 19 million worldwide annually [2], and in Sweden, the incidence is 780/100,000 inhabitants/year [3]. Patients with sepsis are often initially cared for by the ambulance service (AS), and approximately 50 to 75% of patients with sepsis are transported to the emergency department (ED) by the AS [4–6].

The initial care and treatment of patients with sepsis is vital for reducing adverse outcome risks due to inadequate assessments or delayed medical interventions [7]. In other words, early suspicion of sepsis could lead to an assessment that can improve the patient’s prognosis and outcome [7, 8]. Previous studies have indicated that a documented suspicion of sepsis in the AS electronic health records shortened the time until the administration of antibiotics [5, 7], which is important because a delayed time to the start of antibiotics is associated with increased progression to septic shock and increased mortality [9]. Consequently, the assessment of sepsis within the AS is of crucial importance. However, research has indicated a large variation in the proportion of patients with sepsis (from 6 to 36%) that have been detected during ambulance care [5, 7, 10, 11]. Currently, the reason for these variations can only invite speculation. One reason might be that ambulance clinicians (ACs) have limited time with the patient and prioritize taking care of the patient rather than documenting observations in the health records [5]. Another reason is that the identification of patients with sepsis in the AS is difficult, and the identification rate may also dependent on the strategy used by the AC [12].

The literature points to challenges related to the early identification of patients with sepsis. To identify the possibility of sepsis, Edman-Waller et al. [13] suggest combining vital signs with the patient’s own description of symptoms. For example, recent studies have indicated that symptoms such as respiratory difficulty, altered mental status, nausea, diarrhoea and/or vomiting, severe localized pain, muscle weakness, lack of energy, fever and/or chills, were common among patients with sepsis [13, 14]. Other studies found that the use of different sepsis screening tools can increase clinicians’ ability to identify patients with sepsis [15–17]. There are several sepsis-related screening tools that have been developed for the assessment of patients during ambulance care [11, 18], such as the PREhospital Severe Sepsis (PRESS) score, Robson screening tool, Sepsis Alert protocol, quick Sequential (Sepsis-related) Organ Failure Assessment (qSOFA) and BAS 90–30-90 (based on oxygen saturation, respiratory rate and systolic blood pressure) [1, 11, 16, 17, 19]. However, these screening tools are limited in validity and efficiency [15, 20]. Another problem with these screening tools could be that they are usually based on vital signs and that they are only used when AC already suspects sepsis based on other information.

To identify patients with sepsis at an early stage, the assessment process among these patients is important. More knowledge about the assessment is needed to lessen the time from signs and symptoms to treatment. It is therefore important to examine ACs’ experiences in assessing patients with suspected sepsis and to identify the relevant signs and symptoms that indicate sepsis. Based on our knowledge, there is no previous qualitative research from the ACs’ perspective on the assessment process of patients with suspected sepsis. To gain in-depth knowledge regarding ACs’ experiences of assessment, the aim of this study was to explore the meaning of ACs’ lived experiences in assessing patients suspected of having sepsis.

## Methods

### Study design

This study employed a descriptive qualitative approach. Data were gathered using dyadic [21] and individual semistructured interviews [22] and were analysed with a qualitative thematic analysis based on descriptive phenomenology [23]. This approach was chosen to shed light on the patterns and meanings from ACs’ lived experiences [23].

### Study setting and participants

Three AS organizations were included. One organization is located in southern Sweden and comprises seven stations in a catchment area of approximately 205,000 inhabitants, and 30,000 annual missions. The other two organizations are located in western Sweden. One comprises nine stations in a catchment area of approximately 264,000 inhabitants, and 36,500 annual missions. The other compromises nine stations in a catchment area of approximately 300,000 inhabitants, and 38,444 annual missions. The ACs are aided by regional guidelines based on the Advanced Medical Life Support concept [24]. In addition to regional guidelines, the AC also uses a triage system called the Rapid Emergency Triage and Treatment System (RETTS). This system is based on assessing vital signs (blood pressure, heart rate, respiratory rate, oxygen saturation, degree of consciousness and body temperature) and Emergency Symptoms and Signs (ESS; assessment of the patient’s condition and medical severity based on anamnesis, symptoms experienced and signs of illness or injury). The assessment with RETTS is based on a combination of vital signs and ESS, resulting in one of five triage colours that defines the designation of priority for an assessment by the physician when entering the ED. The triage colour red stands for life threatening, orange for potentially life threatening, yellow and green mean ‘can wait’, although yellow is considered to require greater urgency than green and the colour blue mean can be managed at a lower level of care than at the ED. [25]

The AS in Sweden is operated by an ambulance team that consists of a combination of one registered nurse (RN) and one emergency medical technician (EMT) or, alternatively, of two RNs [26, 27]. EMT responsibilities include driving the ambulance and being part of the team that forms the first link in prehospital emergency care. An EMT is a three-year upper school educated nurse assistant with a supplementary 0.5–1 year of education in prehospital emergency care [28]. The RN has the main responsibility for patient care, and based on regional guidelines and general delegation, the RN also independently administers approximately 30 different drugs as needed. In Sweden, RNs’ education includes 3 years of university studies, leading to a Bachelor of Science degree in Nursing. Some RNs have a specialist nursing degree. The Specialist Nurse Programme for prehospital emergency care in Sweden corresponds with 60 credits in the European Credit Transfer System, which are taken through a postgraduate program for RNs that results in a one-year Postgraduate Diploma in Specialist Nursing Prehospital Emergency Care and a Master of Science degree with a major in Nursing Science [27]. In this study, all personnel involved in ambulance care were labelled ACs.

Written approval to perform the interviews was obtained from the Head of Department responsible for the AS organizations. ACs were then informed about in the study by the Head of Unit at a staff meeting or through the workplace’s website. Those who met the inclusion criteria contacted or were contacted by the researcher if they were interested in being interviewed. Two of the contacted ACs declined the request due to lack of time. The inclusion criteria for AC were as follows: having two or more years of work experience in AS, having cared for patients with sepsis within the AS and wanting to talk about their experiences of suspected sepsis. In total, 19 ACs participated. They were aged 25 to 51 years and had been working in the AS for 2 to 25 years. Among the participants, 2 were EMTs, and 17 were RNs; of those, 14 had a specialist nursing degree.

### Data collection

The interviews were conducted from September to November 2019 by the first author who had training in qualitative research by doctoral courses and working experience in the AS. In total, 14 face-to-face interviews were conducted, including five dyadic interviews and nine individual interviews. Repeated interviews with the same participants were not conducted. The ACs were given the choice of whether they wanted to participate individually or together with another AC with whom they had worked closely. All interviews were digitally recorded and transcribed verbatim by the first author. The first three interviews were transcribed immediately after the interviews were conducted and were read through by the third and last authors to ensure that the interview guide and process were effective. Both authors agreed that the interview guide was appropriate and effective, and no adjustments were made. The dyadic interviews took 41–75 min, and the individual interviews took 45–66 min. The total interview time was 12 h and 3 min. The ACs chose the time, day and place for their interviews and were off duty during the interview. The interviews were conducted at the participant’s workplace, and no one else was present other than the participant and the interviewer. Data were collected through semistructured interviews with open-ended questions [22]. The questions from the interview guide were as follows: Can you tell us about your experiences to suspect patients with sepsis in the AS? Can you tell us about what in your assessment causes you to suspect sepsis in the AS? Can you reason about what you experience as difficulty when assessing suspected sepsis in the AS? Can you reason about what you experience as easy when assessing suspected sepsis in the AS? Can you tell us about a case where you suspected a patient of having sepsis in the AS? Open-ended follow up questions were asked when appropriate e.g.: Can you tell me more about that? What do you mean by that? to gain more detailed descriptions of the ACs experiences.

### Data analysis

Qualitative thematic analysis based on descriptive phenomenology was used [23]. The analysis started with reading the interviews repeatedly so that a sense of the wholeness of the data was obtained. After repeated reading of the interviews, meanings were highlighted that responded to the aim. Notes and short descriptive words were used to give meanings preliminary names. As the analysis progressed, meanings that were related to each other were compared to identify differences and similarities. Meanings that were related to each other were organized into patterns. Patterns were successively identified and then grouped into initial themes. The themes were then reviewed and more carefully defined and renamed as the analysis progressed. The analysis was mainly performed by the first author and discussed and validated on several occasions with the second and last authors to reach agreement. Finally, four themes were found to capture the meaning of the ACs’ lived experiences assessing patients suspected of having sepsis. These themes and the result in its entirety were discussed and validated among all authors.

All authors have worked as RNs and thus have an understanding of assessing patients with suspected sepsis. The first, second and fourth authors are also ACs who have worked in the AS. All of the authors have a contextual preunderstanding of the research field. Therefore, the authors need to sustain reflexivity during the entire analysis process. As qualitative researchers are closely engaged in the research process, they must reflect on what the data actually state, which may be different from the researchers’ understanding. This means that the researcher should question the findings instead of taking them for granted [23]. The questioning of our pre-understanding was carried out by highlighting and discussing our own view of assessing patients with suspected sepsis throughout the analysis process. Quotations from different participants are also presented in the results, which could enhance the credibility by enabling the reader to decide whether or interpretations are reasonable [23].

### Ethical considerations

This type of study is not within the boundaries of the ethics review act 2003:460 which regulates all types of research involving humans in Sweden [29]. The research was, however, conducted in accordance with the requirements of the Helsinki declaration [30]. This was done by ensuring that all participants were treated with respect for autonomy, beneficence, nonmaleficence and justice. Verbal and written information about the study was provided and all participants gave their written informed consent. AC who participated were ensured of confidentiality and their right to withdraw at any time without giving any explanation.

## Results

**Fig. 1:**
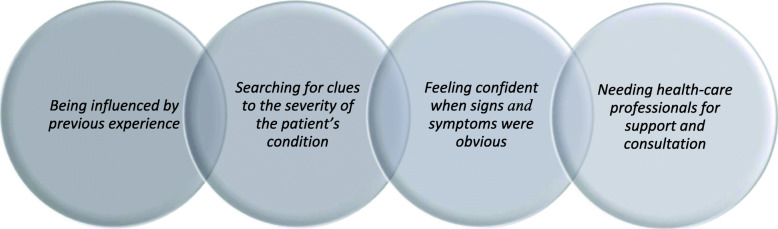
Themes describing AC experiences of the assessment when suspecting patients with sepsis

The lived experiences of assessing patients with suspected sepsis are described based on four themes (see Fig. 1). These themes can be understood as parts that are more or less combined, e.g., to identify patients with suspected sepsis, ACs need to combine information gathered from all of these parts. The assessment was influenced by their previous experiences of meeting patients with sepsis, observations of the patients’ course of the disease, and the extent to which their suspicion was supported by guidelines or other health care professionals. The more obvious the signs and symptoms were, the more accurate the ACs became in their assessments of sepsis. Uncertainty tended to arise if signs and symptoms were perceived as vague during the assessment process, making the final decision to determine the choice of treatment and care more difficult.

### Being influenced by previous experience

The impact of one’s experience refers to how previous experience from similar care encounters creates knowledge and influences the assessment. Even though the ACs did not reflect on what their suspicion of sepsis was based on, their previous experience was described as a prerequisite for increasing the possibility of a correct assessment.

*However, through experience, when you have seen many of them [patients with sepsis], you see that there is so much more. However, you cannot see this ‘more’ if you do not know the other. I mean, if you do not understand that a high fever and low saturation can mean something, maybe you are not connecting it with abdominal pain as well.*

Previous experience also contributed to uncertainty in the assessment. This means that previous experience also led ACs to question whether the patient’s health problem was caused by sepsis or was due to something else. This uncertainty was described as being greater when the patient did not exhibit traditional signs or symptoms from checklists for the onset of sepsis. For example, with young patients, difficulty was noted with interpreting signs; the same was found to be true for cases of underlying diseases that may camouflage the patient’s signs and symptoms of sepsis.

*Sepsis may be due to pneumonia. However, shortness of breath and rattling in the lungs can also be heart failure. They two collide. You get low saturation on both and we have no fever. And with a high respiratory rate and high heart rate, it will certainly be difficult to distinguish between them.*

### Searching for clues to the severity of the patient’s condition

Searching for clues means finding indications or warning signs about the serious of the patient’s condition, i.e., that strengthen or weaken the suspicion of sepsis. It could be based on what was narrated but also on what can be measured and observed. A warning sign described was the patient’s story indicating a sudden onset or a sudden deterioration. For example, when the patient expressed “I have never felt so sick”, it was perceived as an important clue indicating something potentially related to sepsis. At the same time, there was an awareness that symptoms were described in different ways. This means that it was a complex task to suspect sepsis based on described symptoms that may also indicate other medical conditions. However, descriptions such as nausea, dizziness, abdominal pain and muscle weakness were recognized as clues that strengthen the suspicion of sepsis.

*On the other hand, if you hear someone tell you that for 3 days it has only gotten worse and worse, and yesterday I could walk but now I just lie in bed and have no strength and I have been taking paracetamol, I have been taking ibuprofen, I have been taking this and this. Here you have a very fast process and it is a little too fast to just be a normal infection.*

Searching for clues also means listening to significant others stories about the patient. Their stories about observed signs and symptoms add important information to the patient’s own story, which leads to increased insight into the severity of the patient’s health condition. This becomes especially important when the patient has a limited ability to explain concerns, e.g., in case a of altered mental status. In these situations, it was valuable to obtain information about whether the patient had been tired, experienced shivering or had a cold that had not passed. It was also helpful to know whether the patient had previously suffered from sepsis.

*It can be significant others’ description of how the development has been. For example, if they can tell that the patient is normally alert and exercising, but is now lying down and cannot answer for himself and generally feels bad. Then, significant others’ descriptions are extremely important.*

Measurements and observations of the patient’s health conditions also increased or decreased the suspicion of sepsis during the assessment. For example, ACs considered high or low body temperature, high respiratory rate, low oxygen saturation, low systolic blood pressure and high serum glucose to be important. Other important signs were respiratory patterns, altered mental status or deviating skin colour. The more warning signs detected, the more easily sepsis was suspected. The ACs also observed the environment surrounding the patient. Environmental signs could include unpleasant odours, lack of household cleaning, unopened postal items or pharmaceutical packs of antipyretic medicines.

*There is many paracetamol here. They have probably been sick for a while or something has happened here that has made it go fast. So I think that you look at the surroundings in those situations.*

### Feeling confident when signs and symptoms were obvious

There was an ambivalence about the use of guidelines. This means that obvious signs and symptoms that correspond to the descriptions in the guidelines confirm sepsis suspicions and provide relief and confidence.

*You can almost exhale when you have someone who has a bunch of deviating parameters, along with elevated body temperature. Wonderful, this is a sepsis, this is an incipient sepsis, now we are on the right track. And I think that is very pleasant and nice to feel.*

However, the guidelines were also described as narrow because they mostly confirmed sepsis when it seemed obvious and the patient was seriously ill. The ACs questioned the usefulness of the guidelines and did not generally receive support from these guidelines for the assessment process.

*It is slightly too rough. I have experienced that when they are truly bad, it is so obvious, so then you would hardly have needed the sepsis program because you would have understood it anyway.*

### Needing health-care professionals for support and consultation

Needing support and consultation from other health-care professionals was described as creating security in the assessment process. When not supported by others, there was an underlying fear of making mistakes. Obtaining information from the dispatch centre was also perceived as preparatory support. Sometimes the dispatch centre could give information about the patient having an elevated body temperature and respiratory rate or an altered mental status. Such information was described as important for the assessment, especially if other health-care professionals made the assessment. At the same time, there was caution about being over reliant on the dispatch centre, since such information received in previous patient cases did not always agree with what was later shown in the meeting with the patient.

*It is written that they have a high fever, that they have a high respiratory rate, and this is something you have in the back of your mind when you enter. It is not always true, but you have it in the back of your mind, and if it is true, then it has helped us.*

The team colleague who is present during the ambulance mission relies on support and consultation during the assessment of the patient. This means that a functioning interaction provides an open and reflective dialogue with the colleague about their view of the condition, as this view has an important role in determining the choice of treatment and care. When team collaboration did not work, ACs with the main responsibility for the patient felt lonely, abandoned and insecure, making it more difficult for them to make decisions about the patient’s condition.

*You are still two in place, so in some way, you can reason together. So you are not the only caregiver who has to sit and figure out something, because hopefully, two think even better than one.*

Needing consultation and support from health-care professionals in home care can provide descriptions about the patient and the patient’s condition and may confirm whether ACs are on the right track or not. Descriptions are particularly important in situations where the patient is unable to independently account for his or her situation. Descriptions of how vital parameters have developed over time, deteriorations in the patient’s normal condition and a previous medical history were described as being helpful.

*He had already sounded the alarm in the morning because he wanted to go to bed and he usually never wants to go to bed, he usually wants to be up all day. So the nursing assistants in home care were worried … he does not look truly well, and then they called the registered nurse in home care, and then she had noted, oh he has a very high fever and he is quite affected.*

In some situations, it was possible to consult nurses or physicians at the ED for the assessment, which could reduce the fear and anxiety of ACs making incorrect decisions. This was described as particularly helpful when signs and symptoms were diffuse.

*Often, when we are thinking, we call the hospital in advance and ask and talk to them ... What do you think? Do you see it as a common fever or as a sepsis alarm? And then they usually say sepsis alarm. However, it may not be identified as sepsis in its true sense.*

## Discussion

This study found the experiences of ACs to influence the assessment and their suspicion of a patient having sepsis. This is in line with a previous study with RNs working in the emergency department, which found that RN experience was considered important in recognizing sepsis. In addition, vital signs alone were not sufficient to identify patients with sepsis [31]. Previous research indicates that more experienced ACs have developed their ability to make assessments of patient signs and symptoms that remind them of previous similar patients [32–34]. Such experience could help identify sepsis at an earlier stage. However, there is also a risk that experienced ACs will decide the cause of the patient’s condition too quickly, which may lead to an incorrect assessment [33, 35].

The results show that the assessment may be complex, as ACs had difficulties specifying particular signs or symptoms that make them suspect sepsis. Instead, they needed to gather information about different signs and symptoms. Based on a broad overall picture, they could form their assessments and suspect patients with sepsis. According to previous studies, sepsis is described as difficult to identify [13, 36]. There may seldom be a single sign or biomarker used to identify sepsis [36]. Therefore, it is important to have an openness towards the patient’s condition. When making an assessment, ACs need to be responsive to the patient’s life story and perceived health condition along with various signs [37]. When patients and significant others trusted the ACs, they were more cooperative and shared more accurate information [32]. Thus, ACs also need to have an open approach and must try to understand the perspective of patients and descriptions from significant others [32].

The results also showed that the assessment was difficult when signs and symptoms were vague and not confirmed by the guidelines. A previous study reported that ACs, regardless of the patient’s condition, assess retrieval equally, and the differences between identifying patients with sepsis or not may lie in the fact that the unidentified patients probably had less clear changes in vital parameters [10]. This may indicate that ACs need more knowledge to improve their understanding and interpretation of vague signs and symptoms related to sepsis and to follow guidelines less strictly. According to Andersson et al. [32], guidelines, in general, may control the assessment too strictly, and thus, they are not easily applicable to a given AS context. However, guidelines have been shown to be valuable in assessing patients in the AS, as they can help ACs avoid making cognitive decision-making errors in the assessment process [38]. Difficulties in assessing patients when signs and symptoms are vague could also indicate that guidelines are designed according to the redefined definition of sepsis, where ongoing organ dysfunction caused by an infection should appear. Thus, the patient shows obvious signs and symptoms of organ dysfunction with an increased risk of mortality [15]. It would be beneficial to have screening tools for detecting patients with sepsis even before organ dysfunction occurs.

The findings described how ACs consulted other healthcare professionals during their assessment. Research has shown that when ACs hesitate in their assessment, they seek confirmation from others, discussing their view with colleagues or other healthcare professionals to find a suitable course of action [32, 37]. Through discussion and feedback with colleagues or other health care professionals, ACs improve and develop their knowledge and skills, thus providing their patients with suitable care [32]. In another study, RNs in the ED were found to seek input from more senior and experienced clinicians to obtain advice about identifying sepsis. They experienced seeking advice as empowering and improving patient safety [31]. This may imply that when guidelines do not confirm an assessment, the ACs searched for warning signs that could indicate patients having sepsis. ACs may need feedback on their assessment and decision-making to develop their knowledge and experience in identifying patients with sepsis. Since ACs work with limited collegial support, education and regular training may be helpful to enable ACs to increase their confidence in assessments and decision-making [32]. There may be a need for increased support through telemedicine between ACs and medical experts when making assessments of patients with suspected sepsis. Telemedicine has been shown to improve the assessment and care of other health conditions, such as stroke and acute myocardial infarction patients in AS patients [39, 40].

### Limitations and strengths

A strength of this study is that both dyadic interviews and individual interviews were conducted, which contributes to the richness of the data. Both dyadic and individual interviews are suitable for collecting data on a deeper and more detailed level compared to focus groups interviews [21]. Letting the participants choose between being interviewed individually or in dyads was assumed to stimulate their willingness to participate and share experiences in forms that suited the participants. Furthermore, the combination of the two methods was judged to contribute with even more variations of experiences. To create a comfortable interaction in dyad interviews, participants need to feel comfortable sharing their experiences with each other [21, 41]. To minimize the risk that the results reflected the AC’s general care experiences, they were asked during the interview to provide concrete examples related to their lived experiences of patients with sepsis. When participants seemed to describe attitudes in general, such descriptions were removed during the analysis. The analysis was discussed among the authors to ensure that the findings were derived from the data [23] and not the authors preunderstanding or preconceptions. The transferability could be limited for other settings since the findings are not demographically representative for all Swedish ACs. Another limitation may be that only two EMTs participated in the study, while most participants were RNs; therefore, the meanings are more reflective of RNs’ perspectives.

## Conclusions

The results of this study show that ACs need to be observant of information and warning signs in the patient’s environment during assessment to suspect sepsis. Sepsis was considered difficult to suspect solely based on guidelines or specific symptoms and signs. The assessment was a complex process requiring a reflective and open stance. This may be particularly important for ACs when entering the patient’s home to capture information regarding patients with vague signs and non-specific symptoms, as information may otherwise be missed. ACs previous experiences seemed to be pivotal during assessment. Feedback after the ambulance mission and discussions with colleagues could support further development of ACs’ professional knowledge as well as support them in future assessments of patients with sepsis.

## Data Availability

The datasets used and analysed during the current study are available from the corresponding author on reasonable request.

## Abbreviations

AC: Ambulance clinician
AS: Ambulance service
ED: Emergency department
EMT: Emergency medical technician
ESS: Emergency Symptoms and Signs
RETTS: Rapid Emergency Triage and Treatment System
RN: Registered nurse
